# Crystal structure and Hirshfeld surface analysis of 2-amino-5-bromo-1,3,4-triazol-3-ium chloride monohydrate

**DOI:** 10.1107/S2056989026004378

**Published:** 2026-04-29

**Authors:** Batirbay Torambetov, Mehmet Akkurt, Khudayar I. Hasanov, Gizachew Mulugeta Manahelohe, Jamshid Ashurov, Shakhnoza Kadirova

**Affiliations:** a National University of Uzbekistan named after Mirzo Ulugbek 4 University St Tashkent 100174 Uzbekistan; b Department of Physics, Faculty of Sciences, Erciyes University, 38039 Kayseri, Türkiye; c Azerbaijan Medical University, Scientific Research Centre (SRC), A. Kasumzade St. 14, AZ 1022, Baku, Azerbaijan; d Department of Chemistry, University of Gondar, PO Box 196, Gondar, Ethiopia; e Institute of Bioorganic Chemistry, Academy of Sciences of Uzbekistan, M. Ulugbek, St, 83, Tashkent, 100125, Uzbekistan; University of Hyogo, Japan

**Keywords:** crystal structure, hydrogen bonds, van der Waals inter­actions, Hirshfeld surface analysis

## Abstract

In the title salt, C_2_H_3_BrN_3_S^+^·Cl^−^·H_2_O, the cation C_2_H_3_BrN_3_S^+^ cation, the Cl^−^ anion and the water mol­ecule are linked by N—H⋯Cl, N—H⋯O and O—H⋯Cl hydrogen bonds, forming mol­ecular layers parallel to the (002) plane. The crystal packing is further reinforced by van der Waals inter­actions between these layers but C—H⋯π and π–π inter­actions are not observed. Hirshfeld surface analysis and two-dimensional fingerprint plots were used to study the inter­molecular inter­actions.

## Abstract 2

In the title salt, the cation (C_2_H_3_BrN_3_S)^+^, the anion Cl- and the water mol­ecule are bonded by N—H⋯Cl, N—H⋯O and O—H⋯Cl hydrogen bonds, forming mol­ecular layers parallel to the (002) plane.

## 1. Chemical context

Thia­diazole is a five-membered ring system containing a chalcogen-bond-donor sulfur atom (Gurbanov *et al.*, 2023 ▸), a hydrogen-bonding domain, and a two-electron donor nitro­gen system that exhibits a strong coordination ability (Khojabaeva *et al.*, 2025 ▸; Mahmudov *et al.*, 2021 ▸). The 1,3,4-thia­diazole moiety is present as a core structural component in an array of drug categories such as anti-inflammatory, anti­microbial, analgesic, anti­cancer, anti-epileptic, anti­neoplastic, anti­viral, and anti­tubercular agents (Jain *et al.*, 2013 ▸; Torambetov *et al.*, 2026 ▸). Transition-metal complexes of thia­diazole ligands have also attracted much attention due to their high synthetic potential for synthesis, crystal engineering and catalysis (Mamedov *et al.*, 2006 ▸; Nuralieva *et al.*, 2025 ▸, 2026 ▸). Function­alization of the thia­diazol moiety with a non-covalent bond-donor or acceptor sites can be used a synthetic tool to improve their functional properties (Huseynov *et al.*, 2021 ▸; Naghiyev *et al.*, 2023 ▸).
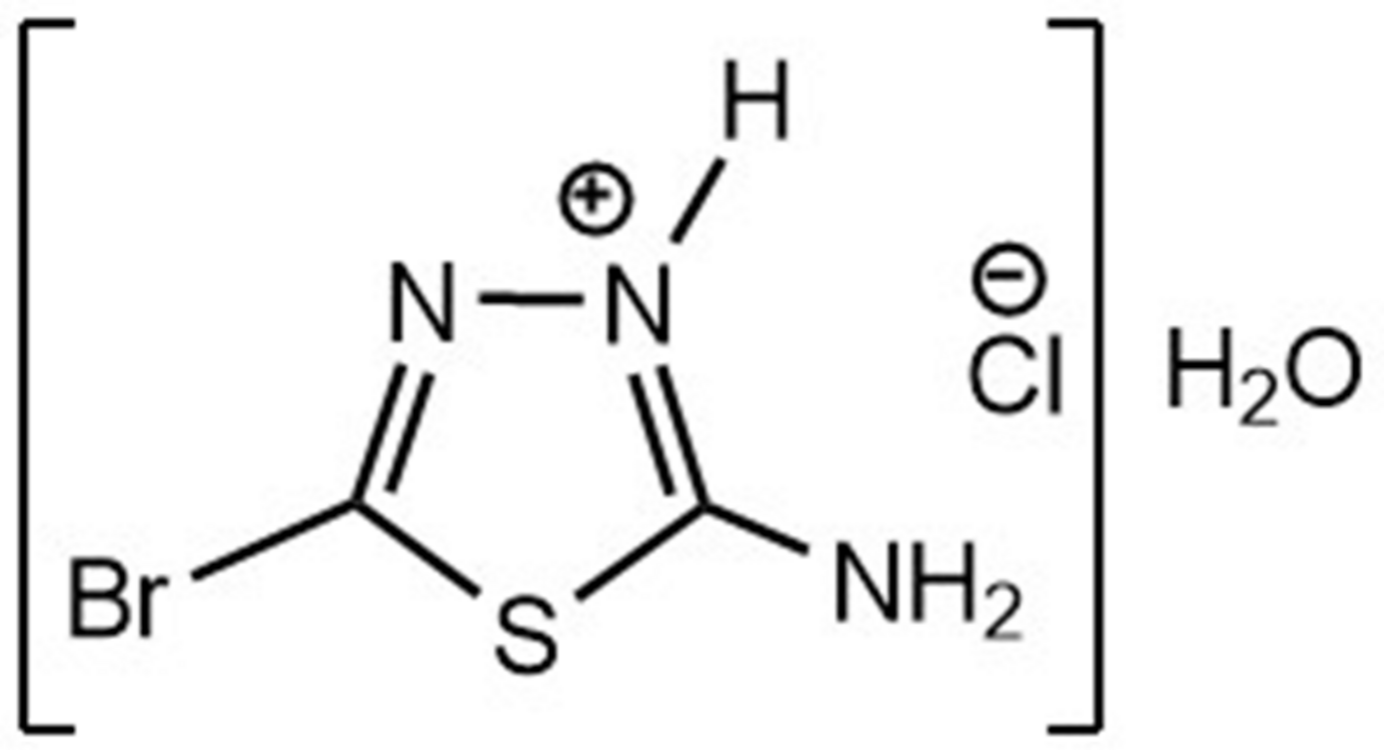


In a continuation of our work in this area, we functionalized a thia­diazol, 5-bromo-1,3,4-thia­diazol-2-amine, which exhibits various sorts of inter­molecular non-covalent inter­actions.

## 2. Structural commentary

**Figure 1:**
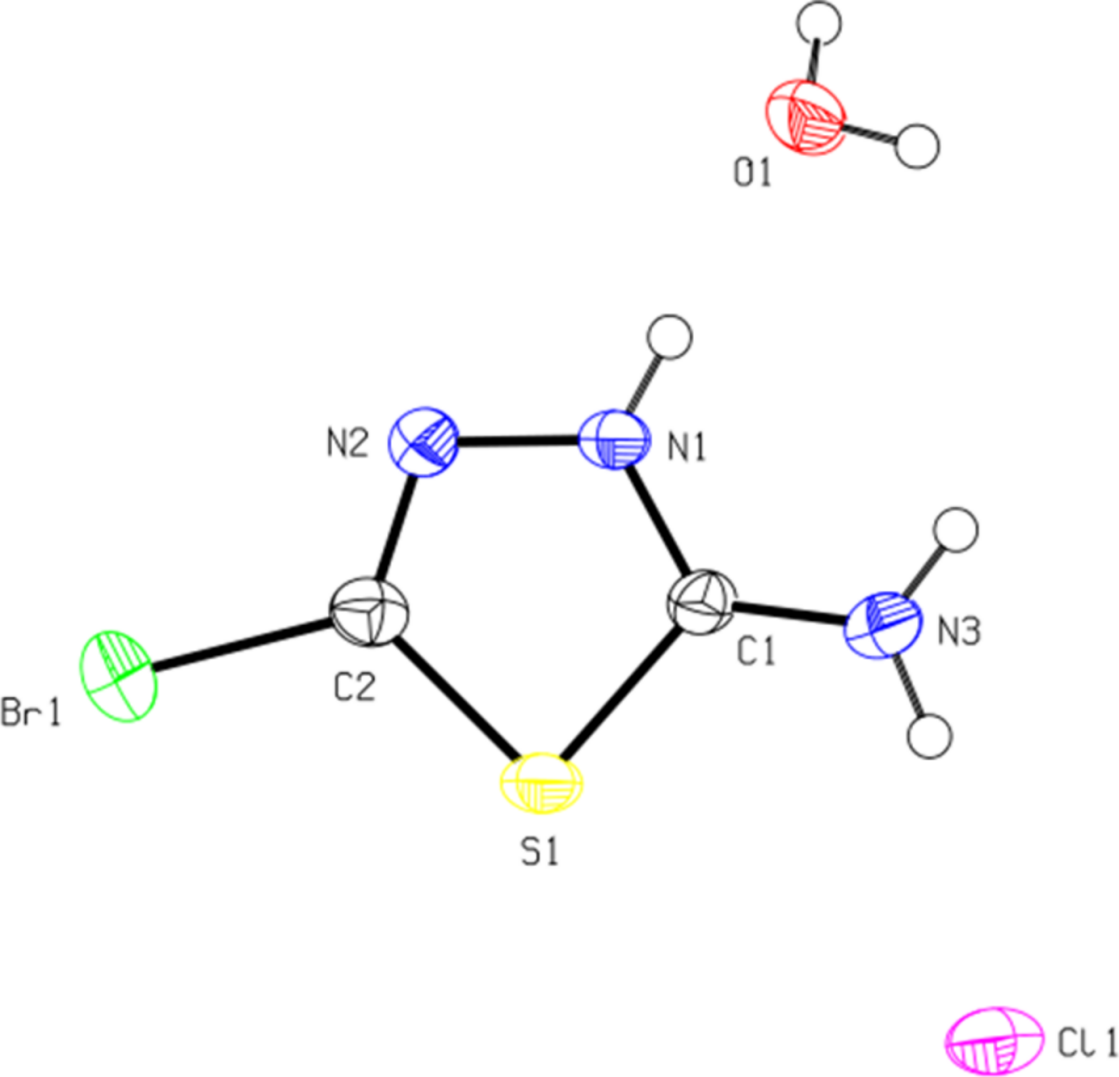
View of the asymmetric unit the title salt, showing the atom labeling and the 50% probability ellipsoids for non-hydrogen atoms.

In the (C_2_H_3_BrN_3_S)^+^ cation of the title salt, (Fig. 1 ▸), the five-membered ring is quite planar (r.m.s. deviation = 0.004 Å). The N1—N2—C2—Br1 and C1—S1—C2—Br1 torsion angles are −179.2 (3) and 178.6 (3)°, respectively. The Br—C [Br1—C2 = 1.855 (5) Å], S—C [S1—C1 = 1.733 (5) and S1—C2 = 1.740 (5) Å], N=C [N1—C1 = 1.323 (6) and N2—C2 =1.277 (7) Å] and N—N [N1—N2 = 1.364 (6) Å] bond lengths, and the C—S—C [C1—S1—C2) 86.9 (2)°] and C=N—N [C1=N1—N2 = 117.5 (4) and C2=N2—N1 = 108.9 (4)°] angles are within normal values and are also compatible with those of the structures in the database survey section.

## 3. Supra­molecular features and Hirshfeld surface analysis

**Figure 2:**
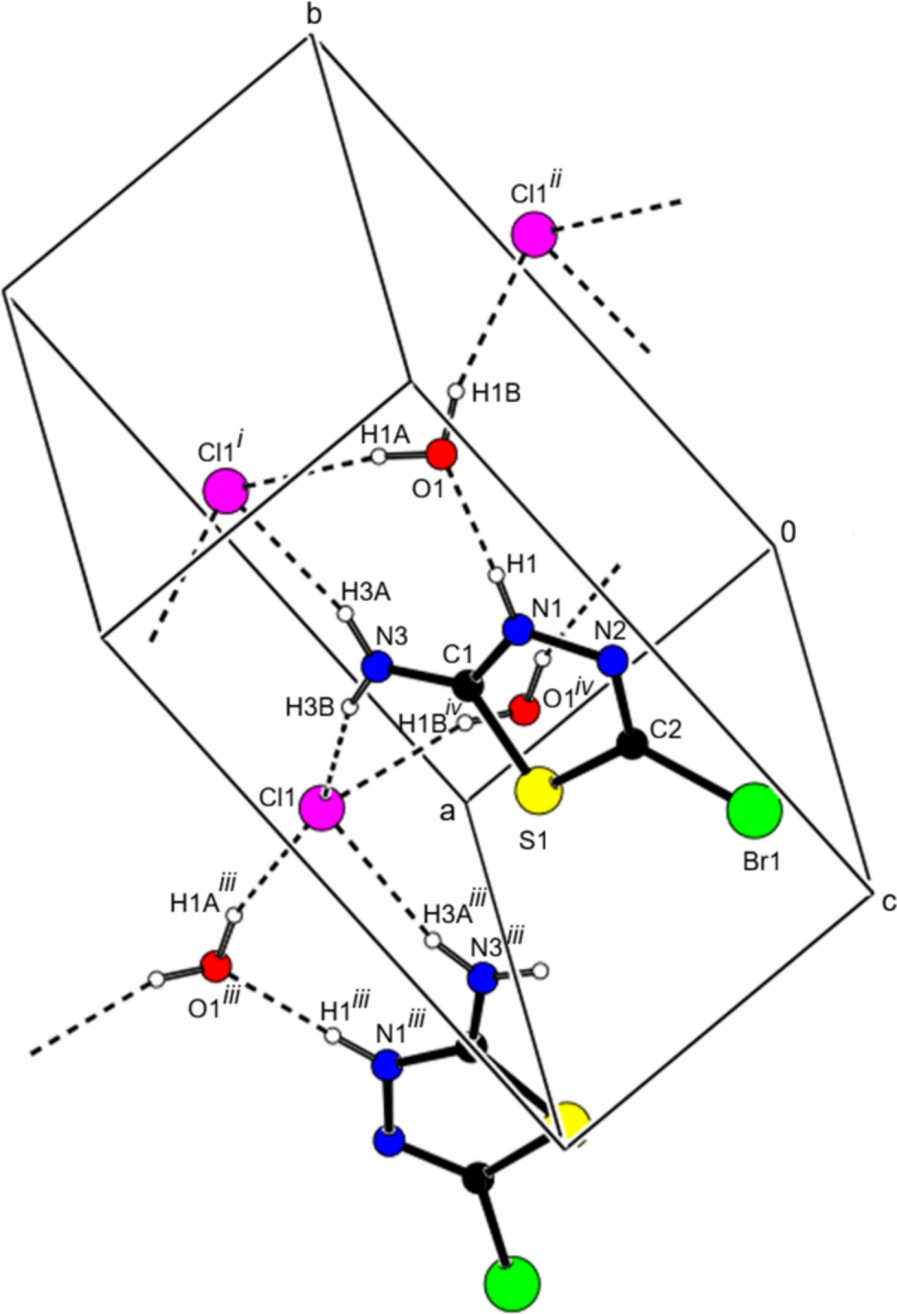
A partial packing diagram showing the O—H⋯Cl, N—H⋯Cl and N—H⋯O hydrogen bonds (dashed lines). Symmetry codes: (i) 2 − *x*, 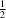 + *y*, 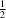 − *z*; (ii) −1 + *x*, *y*, *z*; (iii) 2 − *x*, −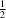 + *y*, 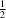 − *z*; (iv) 1 − *x*, −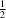 + *y*, 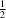 − *z*.

In the title salt, the (C_2_H_3_BrN_3_S)^+^ cation, the Cl^−^ anion, and the water mol­ecule are linked by N—H⋯Cl, N—H⋯O, and O—H⋯Cl hydrogen bonds, forming mol­ecular layers parallel to the (002) plane (Table 1 ▸, Figs. 2 ▸, 3 ▸ and 4 ▸). van der Waals inter­actions between these layers consolidate the crystal packing. C—H⋯π and π–π inter­actions were not observed.

**Figure 5:**
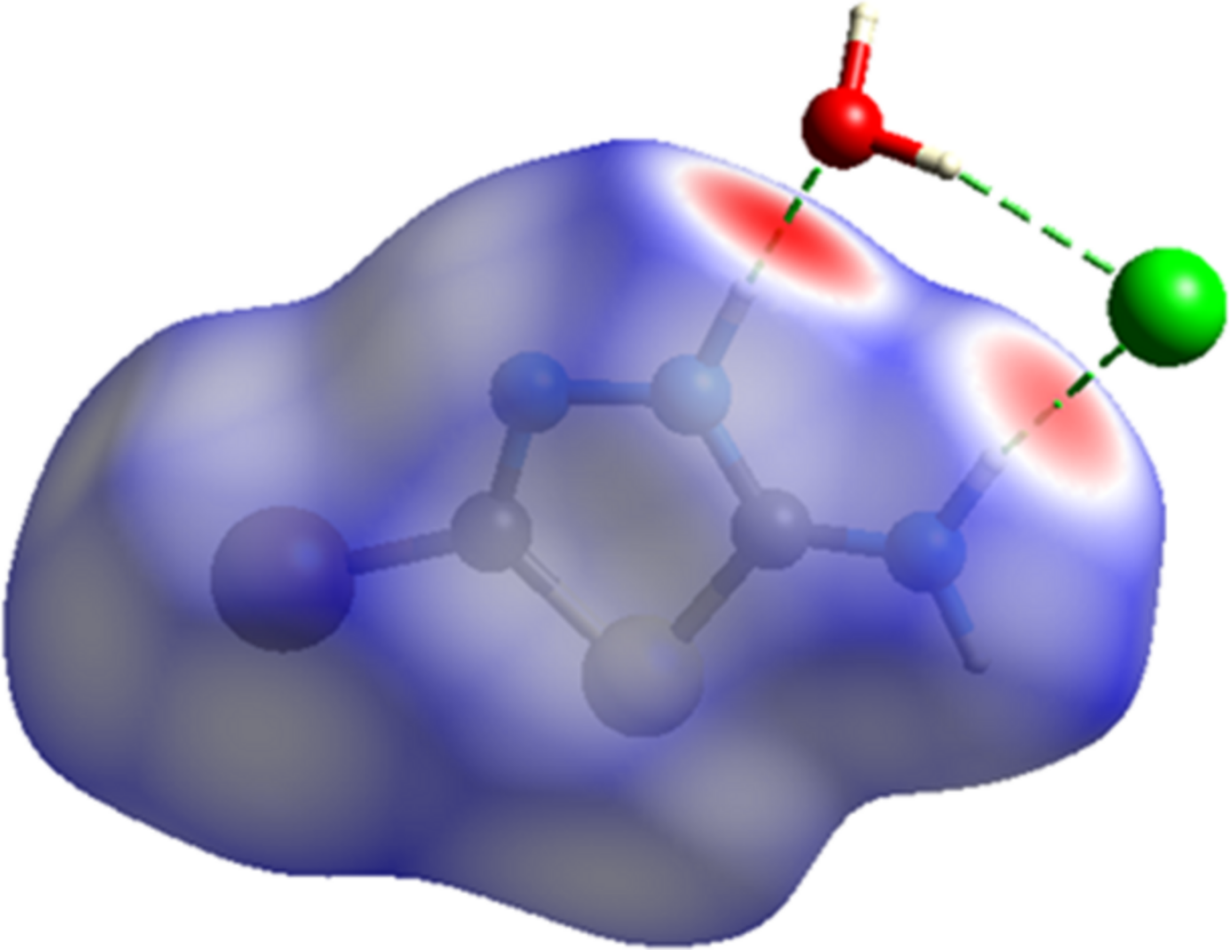
The Hirshfeld surface of the (C_2_H_3_BrN_3_S)^+^ cation of the title salt mapped over *d*_norm_ (color code. Br: green, C: gray; Cl: violet, H: white; O: red; N: blue; S: yellow).

**Figure 6:**
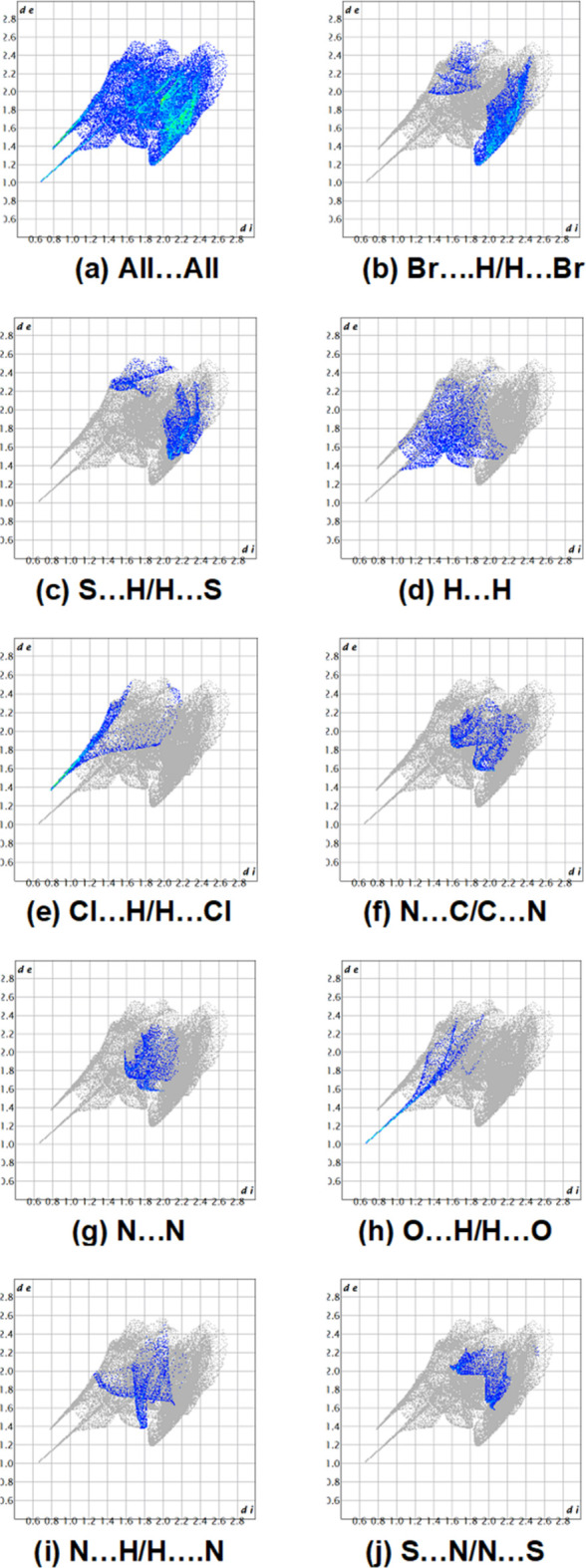
The two-dimensional fingerprint plots, showing (*a*) all inter­actions, and those delineated into (*b*) Br⋯H/H⋯Br, (*c*) S⋯H/H⋯S, (*d*) H⋯H, (*e*) Cl⋯H/H⋯Cl, (*f*) N⋯C/C⋯N, (*g*) N⋯N, (*h*) O⋯H/H⋯O, (i) N⋯H/H⋯N and (*j*) S⋯N/N⋯S inter­actions; *d*_e_ and *d*_i_ represent the distances from a point on the Hirshfeld surface to the nearest atoms outside (external) and inside (inter­nal) the surface, respectively.

Hirshfeld surface analysis was performed to visualize and qu­antify the inter­molecular inter­actions in the cation of the title salt using *CrystalExplorer* (Spackman *et al.*, 2021 ▸). The Hirshfeld surfaces were mapped over *d*_norm_ in the range −0.7220 (red) to +0,9614 (blue) a.u. (Fig. 5 ▸). The red regions are attributed to the N1—H1⋯O1 and N3—H3B⋯Cl1 inter­actions (Tables 1 ▸ and 2 ▸). The two-dimensional fingerprint plots indicate that the major contributions to the crystal packing are from Br⋯H/H⋯Br (21.4%), H⋯H (9.6%) and Cl⋯H/H⋯Cl (7.5%) inter­actions as shown in Fig. 6 ▸. Other, less notable contacts are from N⋯C/C⋯N (5.5%), N⋯N (5.3%), O⋯H/H⋯O (5.2%), N⋯H/H⋯N (5.1%), S⋯N/N⋯S (4.6%), Br⋯S/S⋯Br (4.3%), Br⋯O/O⋯Br (4.1%), C⋯H/H⋯C (4.1%), Cl⋯S/S⋯Cl (3.1%), Br⋯Cl/Cl⋯Br (2.2%), Cl⋯C/C⋯Cl (2.0%), Br⋯N/N⋯Br (1.8%), Cl⋯N/N⋯Cl (1.2%), S⋯O/O⋯S (0.8%), S⋯C/C⋯S (0.7%), Br⋯·C/C⋯Br (0.3%), C⋯C (0.1%) and O⋯C/C⋯O (0.1%) inter­actions.

## 4. Database survey

A search of the Cambridge Structural Database (CSD, Version 6.00, last update April 2025; Groom *et al.*, 2016 ▸) for the cation without the Br atom gave one hit and this is a copper complex. When the unprotonated mol­ecule is searched for by removing Br, 240 results are obtained, and most of these are metal complexes. The three most similar compounds to the title salt containing the 2-amino-5-bromo-1,3,4-triazol-3-ium unit are CSD refcodes AYOVAM (Zhang *et al.*, 2011 ▸), VIKSOZ (Smith & Lynch, 2013 ▸) and BOMROM (Smith & Lynch, 2014 ▸).

In AYOVAM, the strongest N—H⋯N inter­molecular hydrogen bond, between the amine group and one thia­diazole N atom, forms centrosymmetric dimers. The other amine H atom extends the supra­molecular network, forming an N—H⋯N contact with the other thia­diazole N atom. In VIKSOZ, the amine-heteroatom N—H⋯N hydrogen bond between the heterodimers results in a one-dimensional chain structure stretching along the *c*-axis direction. In BOMROM, the heterodimers are extended into a chain along the *b*-axis direction through an amine N—H⋯N thia­diazole hydrogen bond. In the title compound, the crystal packing is achieved through inter­molecular O—H⋯Cl and N—H⋯Cl hydrogen bonds.

## 5. Synthesis and crystallization

To a solution of 2-amino-1,3,4-thia­diazole (5 g, 48.45 mmol) in methanol (70 mL), sodium bicarbonate (8.14 g, 96.90 mmol) and bromine (2.5 mL, 48.45 mmol) were added. The reaction mixture was stirred at room temperature until the disappearance of the starting material (30–40 minutes). The methanol was removed under vacuum and the crude product was diluted with water (15 mL), filtered, dry *in vacuo* to give a brown solid, 5-bromo-1,3,4-thia­diazol-2-amine (94%). Colorless crystals suitable for X-ray analysis were obtained by slow evaporation of a mixture of 1 *M* HCl (pH = 0) and methanol (*v*/*v*, 1:2) solution. Analysis calculated for C_2_H_5_BrClN_3_OS (*M* = 234.50): C 10.24, H 2.15, N 17.95; found: C 10.20, H 2.10, N 17.92%. ^1^H NMR (300 MHz, DMSO-*d*^6^): *δ* 7.84 (3H). ^13^C NMR (75 MHz, DMSO-*d*_6_) *δ* 154.8 and 159.1.

## 6. Refinement

Crystal data, data collection and structure refinement details are summarized in Table 3 ▸. The water H-atom positions were determined from the difference-Fourier map, with their thermal characteristics restricted to 1.5 times those of the oxygen atom. The hydrogen atom of the NH group was identified in the difference-Fourier map, refined freely with 1.2*U*_eq_(N), while the hydrogen atoms of the NH_2_ groups were positioned geometrically and assigned thermal parameter values at 1.2 times that of the connected nitro­gen atom.

## Supplementary Material

Crystal structure: contains datablock(s) I. DOI: 10.1107/S2056989026004378/ox2022sup1.cif

Structure factors: contains datablock(s) I. DOI: 10.1107/S2056989026004378/ox2022Isup2.hkl

Supporting information file. DOI: 10.1107/S2056989026004378/ox2022Isup3.cml

CCDC reference: 2549381

Additional supporting information:  crystallographic information; 3D view; checkCIF report

## Figures and Tables

**Figure 3:**
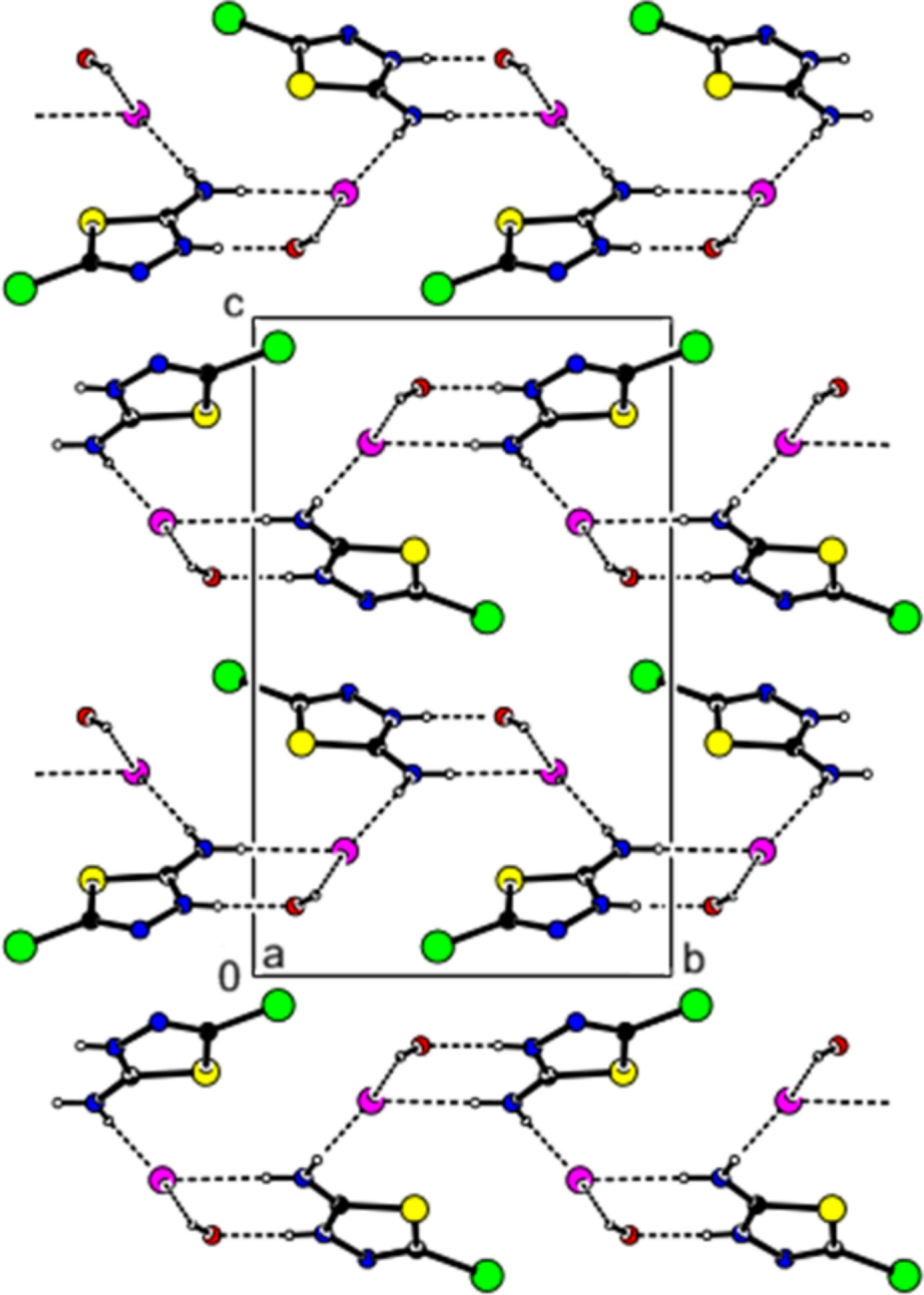
Crystal packing of the title salt viewed along the *a*-axis direction. Inter­mol­ecular hydrogen bonds are shown as dashed lines.

**Figure 4:**
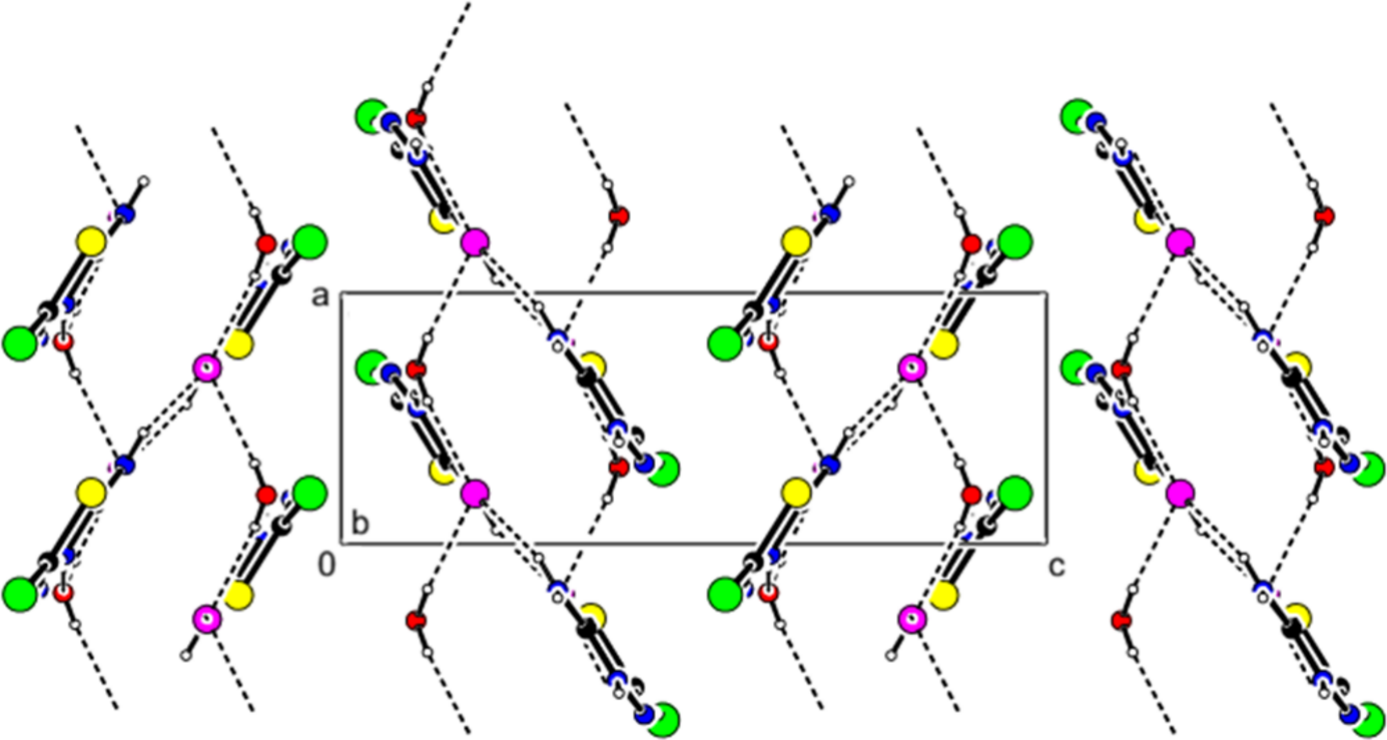
Crystal packing of the title salt viewed along the *b*-axis direction.

**Table 1:** Hydrogen-bond geometry (Å, °)

*D*—H⋯*A*	*D*—H	H⋯*A*	*D*⋯*A*	*D*—H⋯*A*
O1—H1*A*⋯Cl1^i^	0.85	2.32	3.144 (4)	163
O1—H1*B*⋯Cl1^ii^	0.85	2.37	3.196 (4)	163
N3—H3*A*⋯Cl1^i^	0.86	2.38	3.221 (5)	168
N3—H3*B*⋯Cl1	0.86	2.29	3.127 (5)	163
N1—H1⋯O1	0.83 (3)	1.86 (3)	2.685 (6)	176 (7)

Symmetry codes: (i) 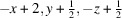; (ii) 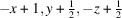.

**Table 2:** Summary of short inter­atomic contacts (Å)

Contact	Distance	Symmetry operation
Br1⋯H1*B*	3.09	*x*, −1 + *y*, *z*
Br1⋯O1	3.50	− 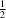 + *x*, 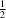 − *y*, 1 − *z*
Br1⋯O1	3.56	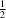 + *x*, 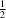 − *y*, 1 − *z*
H1⋯O1	1.85	*x*, *y*, *z*
H3*B*⋯Cl1	2.29	*x*, *y*, *z*
H3*A*⋯Cl1	2.38	2 − *x*, 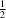 + *y*, 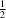 − *z*
C1⋯Cl1	3.51	−1 + *x*, *y*, *z*
Cl1⋯H1*B*	2.37	1 − *x*, − 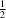 + *y*, 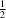 − *z*
Cl1⋯H1*A*	2.32	2 − *x*, − 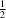 + *y*, 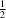 − *z*

**Table 3:** Experimental details

Crystal data	
Chemical formula	C_2_H_3_BrN_3_S^+^·Cl^−^·H_2_O
*M*_r_	234.51
Crystal system, space group	Orthorhombic, *P*2_1_2_1_2_1_
Temperature (K)	293
*a*, *b*, *c* (Å)	5.3575 (1), 9.5328 (1), 15.0588 (2)
*V* (Å^3^)	769.08 (2)
*Z*	4
Radiation type	Cu *K*α
μ (mm^−1^)	12.49
Crystal size (mm)	0.18 × 0.14 × 0.08

Data collection	
Diffractometer	XtaLAB Synergy, Single source at home/near, HyPix3000
Absorption correction	Multi-scan (*CrysAlis PRO*; Rigaku OD, 2020 ▸)
*T*_min_, *T*_max_	0.341, 1.000
No. of measured, independent and observed [*I* > 2σ(*I*)] reflections	6293, 1495, 1485
*R*_int_	0.056
(sin θ/λ)_max_ (Å^−1^)	0.615

Refinement	
*R*[*F*^2^ > 2σ(*F*^2^)], *wR*(*F*^2^), *S*	0.035, 0.097, 1.09
No. of reflections	1495
No. of parameters	90
No. of restraints	5
H-atom treatment	H atoms treated by a mixture of independent and constrained refinement
Δρ_max_, Δρ_min_ (e Å^−3^)	0.63, −0.48
Absolute structure	Flack *x* determined using 579 quotients [(*I*^+^)−(*I*^−^)]/[(*I*^+^)+(*I*^−^)] (Parsons *et al.*, 2013 ▸).
Absolute structure parameter	0.03 (3)

Computer programs: *CrysAlis PRO* (Rigaku OD, 2020 ▸), *SHELXT* (Sheldrick, 2015*a* ▸), *SHELXL* (Sheldrick, 2015*b* ▸), *ORTEP-3 for Windows* (Farrugia, 2012 ▸) and *PLATON* (Spek, 2020 ▸).

## supplementary crystallographic information

### 2-Amino-5-bromo-1,3,4-triazol-3-ium chloride monohydrate Crystal data

**Table:**

C_2_H_3_BrN_3_S^+^·Cl^−^·H_2_O	*D*_x_ = 2.025 Mg m^−^^3^
*M**_r_* = 234.51	Cu *K*α radiation, λ = 1.54184 Å
Orthorhombic, *P*2_1_2_1_2_1_	Cell parameters from 6428 reflections
*a* = 5.3575 (1) Å	θ = 2.9–71.3°
*b* = 9.5328 (1) Å	µ = 12.49 mm^−^^1^
*c* = 15.0588 (2) Å	*T* = 293 K
*V* = 769.08 (2) Å^3^	Block, colourles
*Z* = 4	0.18 × 0.14 × 0.08 mm
*F*(000) = 456	

### 2-Amino-5-bromo-1,3,4-triazol-3-ium chloride monohydrate Data collection

**Table:**

XtaLAB Synergy, Single source at home/near, HyPix3000 diffractometer	1495 independent reflections
Radiation source: micro-focus sealed X-ray tube, PhotonJet (Cu) X-ray Source	1485 reflections with *I* > 2σ(*I*)
Mirror monochromator	*R*_int_ = 0.056
Detector resolution: 10.0000 pixels mm^-1^	θ_max_ = 71.4°, θ_min_ = 5.5°
ω scans	*h* = −5→6
Absorption correction: multi-scan (CrysAlisPro; Rigaku OD, 2020)	*k* = −11→11
*T*_min_ = 0.341, *T*_max_ = 1.000	*l* = −18→18
6293 measured reflections	

### 2-Amino-5-bromo-1,3,4-triazol-3-ium chloride monohydrate Refinement

**Table:**

Refinement on *F*^2^	H atoms treated by a mixture of independent and constrained refinement
Least-squares matrix: full	*w* = 1/[σ^2^(*F*_o_^2^) + (0.0666*P*)^2^ + 0.3887*P*] where *P* = (*F*_o_^2^ + 2*F*_c_^2^)/3
*R*[*F*^2^ > 2σ(*F*^2^)] = 0.035	(Δ/σ)_max_ < 0.001
*wR*(*F*^2^) = 0.097	Δρ_max_ = 0.63 e Å^−^^3^
*S* = 1.09	Δρ_min_ = −0.48 e Å^−^^3^
1495 reflections	Extinction correction: SHELXL-2016/6 (Sheldrick, 2015b), Fc^*^=kFc[1+0.001xFc^2^λ^3^/sin(2θ)]^-1/4^
90 parameters	Extinction coefficient: 0.0048 (8)
5 restraints	Absolute structure: Flack *x* determined using 579 quotients [(*I*^+^)-(*I*^-^)]/[(*I*^+^)+(*I*^-^)] (Parsons *et al.*, 2013).
Primary atom site location: dual	Absolute structure parameter: 0.03 (3)
Hydrogen site location: mixed	

### 2-Amino-5-bromo-1,3,4-triazol-3-ium chloride monohydrate Special details

**Table:**

Geometry. All esds (except the esd in the dihedral angle between two l.s. planes) are estimated using the full covariance matrix. The cell esds are taken into account individually in the estimation of esds in distances, angles and torsion angles; correlations between esds in cell parameters are only used when they are defined by crystal symmetry. An approximate (isotropic) treatment of cell esds is used for estimating esds involving l.s. planes.

### 2-Amino-5-bromo-1,3,4-triazol-3-ium chloride monohydrate Fractional atomic coordinates and isotropic or equivalent isotropic displacement parameters (Å^2^)

**Table:**

	*x*	*y*	*z*	*U*_iso_*/*U*_eq_	
Br1	0.29817 (13)	−0.05893 (6)	0.45541 (4)	0.0458 (3)	
Cl1	1.2003 (2)	0.21834 (13)	0.18983 (8)	0.0381 (4)	
S1	0.7039 (2)	0.11486 (12)	0.35421 (8)	0.0319 (3)	
O1	0.3054 (8)	0.5998 (4)	0.3940 (3)	0.0435 (9)	
H1A	0.431399	0.647416	0.377691	0.065*	
H1B	0.180495	0.648222	0.377514	0.065*	
N1	0.4579 (7)	0.3315 (4)	0.3915 (3)	0.0269 (8)	
N2	0.3207 (8)	0.2273 (4)	0.4298 (3)	0.0288 (8)	
N3	0.8144 (10)	0.3806 (5)	0.3069 (3)	0.0420 (11)	
H3A	0.785060	0.469288	0.306957	0.050*	
H3B	0.943662	0.348223	0.279993	0.050*	
C1	0.6621 (8)	0.2948 (5)	0.3479 (3)	0.0254 (9)	
C2	0.4283 (9)	0.1099 (5)	0.4153 (3)	0.0280 (10)	
H1	0.406 (12)	0.414 (3)	0.394 (4)	0.036 (16)*	

### 2-Amino-5-bromo-1,3,4-triazol-3-ium chloride monohydrate Atomic displacement parameters (Å^2^)

**Table:**

	*U*^11^	*U*^22^	*U*^33^	*U*^12^	*U*^13^	*U*^23^
Br1	0.0516 (4)	0.0277 (4)	0.0581 (4)	−0.0044 (3)	0.0093 (3)	0.0041 (2)
Cl1	0.0264 (5)	0.0377 (7)	0.0501 (7)	0.0046 (5)	−0.0006 (5)	−0.0154 (5)
S1	0.0258 (5)	0.0263 (6)	0.0435 (6)	0.0058 (5)	0.0041 (5)	−0.0054 (5)
O1	0.0295 (17)	0.0276 (19)	0.073 (3)	0.0051 (16)	0.006 (2)	0.0073 (17)
N1	0.0231 (17)	0.022 (2)	0.036 (2)	0.0028 (16)	0.0047 (16)	−0.0036 (15)
N2	0.0259 (19)	0.026 (2)	0.0342 (19)	−0.0007 (17)	0.0043 (16)	−0.0015 (16)
N3	0.036 (2)	0.030 (2)	0.060 (3)	0.001 (2)	0.022 (2)	−0.006 (2)
C1	0.019 (2)	0.024 (2)	0.033 (2)	−0.0016 (17)	0.0011 (17)	−0.0043 (18)
C2	0.026 (2)	0.027 (2)	0.031 (2)	0.001 (2)	−0.0031 (18)	−0.0049 (18)

### 2-Amino-5-bromo-1,3,4-triazol-3-ium chloride monohydrate Geometric parameters (Å, º)

**Table:**

Br1—C2	1.855 (5)	N1—C1	1.323 (6)
S1—C1	1.733 (5)	N1—H1	0.83 (3)
S1—C2	1.740 (5)	N2—C2	1.277 (7)
O1—H1A	0.8500	N3—H3A	0.8600
O1—H1B	0.8501	N3—H3B	0.8600
N1—N2	1.364 (6)	N3—C1	1.309 (7)
			
C1—S1—C2	86.9 (2)	C1—N3—H3B	120.0
H1A—O1—H1B	104.5	N1—C1—S1	110.0 (4)
N2—N1—H1	119 (5)	N3—C1—S1	124.3 (4)
C1—N1—N2	117.5 (4)	N3—C1—N1	125.7 (5)
C1—N1—H1	123 (5)	S1—C2—Br1	121.0 (3)
C2—N2—N1	108.9 (4)	N2—C2—Br1	122.4 (4)
H3A—N3—H3B	120.0	N2—C2—S1	116.7 (4)
C1—N3—H3A	120.0		
			
N1—N2—C2—Br1	−179.2 (3)	C1—S1—C2—N2	−0.6 (4)
N1—N2—C2—S1	−0.1 (5)	C1—N1—N2—C2	1.0 (6)
N2—N1—C1—S1	−1.4 (5)	C2—S1—C1—N1	1.1 (4)
N2—N1—C1—N3	179.6 (5)	C2—S1—C1—N3	−180.0 (5)
C1—S1—C2—Br1	178.6 (3)		

### 2-Amino-5-bromo-1,3,4-triazol-3-ium chloride monohydrate Hydrogen-bond geometry (Å, º)

**Table:**

*D*—H···*A*	*D*—H	H···*A*	*D*···*A*	*D*—H···*A*
O1—H1*A*···Cl1^i^	0.85	2.32	3.144 (4)	163
O1—H1*B*···Cl1^ii^	0.85	2.37	3.196 (4)	163
N3—H3*A*···Cl1^i^	0.86	2.38	3.221 (5)	168
N3—H3*B*···Cl1	0.86	2.29	3.127 (5)	163
N1—H1···O1	0.83 (3)	1.86 (3)	2.685 (6)	176 (7)

Symmetry codes: (i) −*x*+2, *y*+1/2, −*z*+1/2; (ii) −*x*+1, *y*+1/2, −*z*+1/2.
